# Supplementary material for: Identification of photoperiod-induced specific miRNAs in the adrenal glands of Sunite sheep (Ovis aries)
Source: Front Vet Sci. 2022 Jul 22;9:888207. doi: 10.3389/fvets.2022.888207 (PMC9354845; doi:10.3389/fvets.2022.888207)
Supplement: Supplementary file 1 [file Data_Sheet_1.ZIP › Supplementary file/miRDeep2 file.zip!/miRDeep2 file/Know_Structure/SP42A2/oar-mir-496.pdf]

miRBase precursor : oar-mir-496
Total read count : 19
oar-miR-496-5p read count : 5
oar-miR-496-3p read count : 13
remaining reads : 1

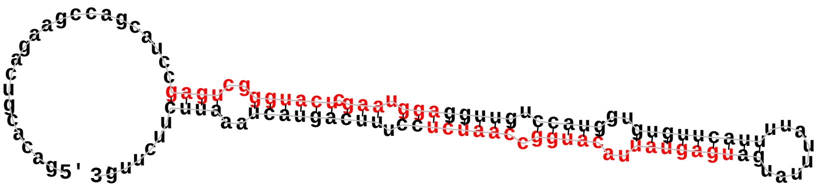

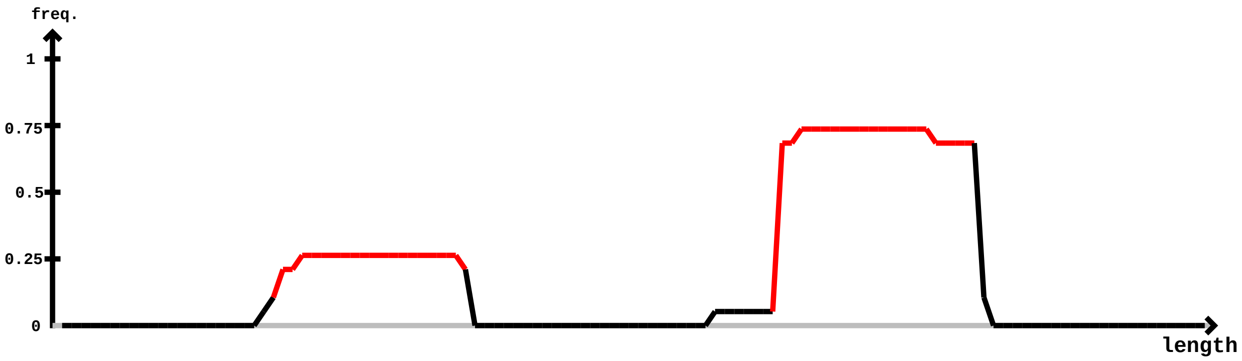


```
                                                                                 oar-miR-496-3p
                     oar-miR-496-5p
5'- gacacgucagaagccagcauccgagucggguacucgaauggagguuguccauggugugucauuuuauuuaugaugaguauuacauggccaaucuccuuucaguacuaaauucuucuug   -3'    exp
    ......................((((..((((((.(((.((((((((.(((((..(((((((((........)))))))))..))))).)))))))).)))))))))..))))......    reads      mm         sam
    ....................ccgagucggguacucgaaugga.............................................................................    1          0          sec
    .....................cgagucggguacucgaaugga.............................................................................    1          0          sec
    ......................gagucggguacucgaaugg..............................................................................    1          0          sec
    ......................gagucggguacucgaaugga.............................................................................    1          0          sec
    ........................gucggguacucgaaugga.............................................................................    1          0          sec
    ...................................................................uuuaugaugaguauuacauggcc.............................    1          0          sec
    ..........................................................................ugaguauuacauggccaaucu........................    11         0          sec
    ..........................................................................ugaguauuacauggccaaucuc.......................    1          0          sec
    ............................................................................aguauuacauggccaaucuc.......................    1          0          sec
```
